# Homoprotocatechuate dioxygenase active site: Imitating the secondary sphere base via computational design

**DOI:** 10.55730/1300-0527.3598

**Published:** 2023-09-30

**Authors:** Muhammed BUYUKTEMIZ, Yavuz DEDE

**Affiliations:** 1 Department of Chemistry, Faculty of Science, Gazi University, Ankara, Turkiye; 2 Department of Chemistry, Faculty of Science, University of Helsinki, Helsinki, Finland

**Keywords:** Enzyme active site, homoprotocatechuate dioxygenase, dioxygenase, proton transfer, secondary sphere

## Abstract

Oxidative ring cleavage reactions have attracted great interest and various studies on the catechol ring-cleaving enzyme homoprotocatechuate dioxygenase (HPCD) have been reported in the literature. The available data on how the proton transfer takes place led us to design a potential HPCD model structure. A secondary sphere effect of utmost importance, the assistance of His200, which is critical for the catechol proton to migrate to dioxygen, was cautiously included on the first coordination shell. This was done mainly by modifying the axial ligands in the first coordination shell of HPCD such that the dual basic/acidic role in the proton transfer pathway of His200 was reproduced. Model systems with mono-, bi-, and tridentate ligands are reported. Energetically feasible reaction channels on synthetically promising ligand structures are identified. Key structural and electronic principles for obtaining viable proton transfer paths are outlined.

## 1. Introduction

**Figure 1:**
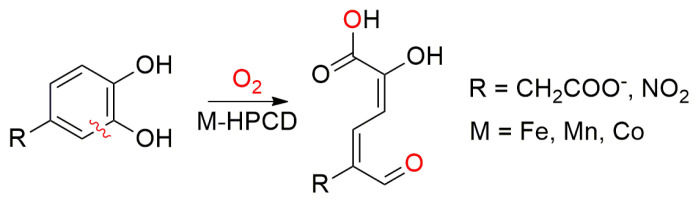
Schematic representation of the cleaving of the catechol derivatives by the HPCD enzyme center.

Aromatic ring cleavage is a challenging reaction that has attracted great attention. Involvement of the triplet diradical dioxygen in these ring cleavage reactions is more attractive, in particular for biological dioxygen reactions. One such reaction is catalyzed by homoprotocatechuate dioxygenase (HPCD), an Fe- or Mn-centered extradiol enzyme that operates in the oxidative ring-opening pathways of aromatic compounds (Figure 1 and Supplementary Figure S1) [1–6].

**Figure 2:**
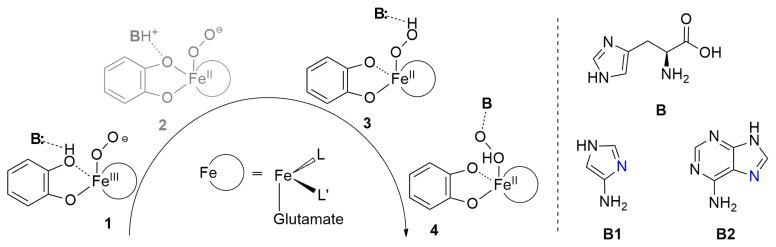
(Left) Proton transfer path studied in this work. (Right) Basic ligands studied here. **B** is present in the native enzyme where L and L’ are histidine residues. For monodentate L (**B1** or **B2** is basic), there is no external **B** in our model. For **B1** and **B2**, L’ is imidazole. **2** is an intermediate in the native enzyme environment.

For Fe–HPCD, comprehensive studies are available in the literature. The spectroscopic data of Lipscomb et al. and computational analyses by Siegbahn et al., Neese et al., and Shaik et al. established a general view about the mechanistic details and nature of many of the intermediates in the HPCD reaction [7–13]. A key structural feature of Fe–HPCD catalysis is the assistance of a secondary sphere His200 residue, denoted below as **B**, in the proton transfer as schematized in Figure 2 [14,15].

His 200 first acts as a Brønsted base and accepts the proton of the catecholate monoanion (**Cat**.) to afford **2** from **1**. The protonated His200 then undergoes a slight change in orientation and interacts with the bound dioxygen to yield **3** or **4**. Direct transfer of the proton to dioxygen is costly as kinetic data on an HPCD mutant showed that the reaction rate is vanishingly small without His200 [14]. If the proton is delivered to the proximal oxygen and **4** is accessed, the distal oxygen becomes highly reactive and attacks the catecholate ring to initiate the cleavage. Apparently, His200 is optimally positioned but also slightly flexible in accomplishing its mission.

Inspired by the role of the His200 residue and noting the importance of mimicking biological reactions, we investigated the proton transfer steps with new ligand environments. The new ligands resemble the catalytic core of Fe–HPCD. The proton-accepting role of the His200 residue of the secondary sphere was reproduced by using a covalently linked amine moiety on the first coordination shell. Among the various internally basic ligand systems considered in this study, synthetic viability was also conceived. Our goal is to propose novel ligand frameworks with the potential to guide further experimental studies of functional models of HPCD. The proton transfer paths were studied using quantum chemical methods and the structural and electronic principles that control the associated energy barriers were delineated.

## 2. Computational details

The proton transfer path given in Figure 2 was studied with density functional theory (DFT) to incorporate the secondary sphere effects of the HPCD active site into the first coordination sphere. Several studies [14,15] have suggested that the His200 of the secondary sphere acts as a proton-transporting agent between the catecholate and O_2_ moieties; ergo, His200 is also responsible for the initiation of the reaction. The proposed structures of the initial steps, following the results of Neese et al. [13], were studied using the UB3LYP/LANL2DZ [16–21] level of theory (Supplementary Figure S2). The dispersion-corrected (Grimme’s D3) [22] M06L density functional [23–25] in combination with the Dunning-type correlation consistent basis set of triple-ζ quality [26] was used for energy evaluations (UM06L-D3/cc-pVTZ). Transition state structures were located using QST approaches along with manual searches when needed. Frequencies were calculated to ensure the intermediates were true minima, i.e., not imaginary frequencies, and that TSs corresponded to first-order saddle points on the potential energy surface. For the transition states, the correspondence of the calculated one and only one imaginary mode to the desired reaction coordinate was confirmed by visual inspection and intrinsic reaction coordinate (IRC) analysis when necessary [27]. Solvation effects were calculated with the PCM model using acetonitrile as the solvent. All calculations were completed using the Gaussian 09 software suite [28]. Of the three possible spin states for addition of the triplet dioxygen to the high-spin Fe(II) center, the intermediate-spin quintet was studied as the reactive surface as discussed in the literature [12,13,29]. This was plausible given the required electronic structure changes on the intrinsic triplet (dioxygen) and quintet (Fe(II)) fragments for bond-breaking and formation as depicted in Supplementary Figure S3. An overall low-spin state restricts the electrons on the Fe center from pairing up, thereby reducing the exchange-enhanced stabilization. The high-spin state, if maintained throughout the reaction, requires the unpairing of lower-lying electrons and hence yields intrinsically excited fragments.

In the interest of exclusion of the secondary sphere, the original ligand system was modified to act as a Brønsted base. Modifications on the axial ligands without disturbing the so-called fac-3 binding motif gave three sets of ligand systems including mono-, bi-, and tridentate ligands. Monodentate ligands utilize imidazole and acetate moieties along with the modified imidazole ring (Supplementary Figures S4 and S5). Two imidazole rings (modified and nonmodified) were further linked with additional CH_2_ groups to increase the rigidity in the bidentate ligand systems while the acetate groups remained untouched (Supplementary Figures S6 and S7). To take synthetic viability into account, tridentate ligands were studied where OAc groups were covalently linked to the ligand system (Supplementary Figures S8 and S9). Details of the structural implications of the ligand systems are discussed thoroughly in Section 3.

Relative energies reported with B3LYP and M06L DFT functionals, structural parameters, spin densities, and Mulliken charges for the selected atoms are given in the supplementary information (Supplementary Tables S1–S5).

## 3. Results and discussion

Proton transfer paths were initially investigated for the monodentate ligands **B1** and **B2** given in Figure 2. Exploration of electronic structure parameters related to reactivity helped us design the bidentate (**B3**, **B4**) and eventually the synthetically promising tridentate (**B5**, **B6**) systems. The intermediate spin is considered to be the productive surface (Supplementary Figure S3) as discussed in the recent literature [12,13,29,30]. Three-dimensional representations of all six ligand environments are provided in the supplementary information (Supplementary Figures S4–S9).

**Figure 3:**
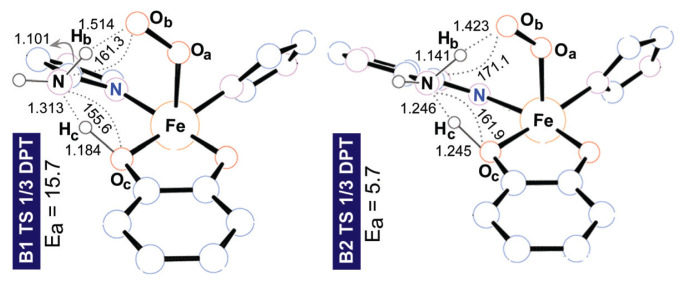
Structural parameters of **TS 1/3 DPT**. Activation energies (E_a_) for **1→3** are calculated as ΔG_(sol)_ and given in kcal mol^−1^.

Monodentate ligands **B1** and **B2** were accompanied by imidazole (L’) and acetate to complete the hexa-coordinate environment of Fe. For the **B1** system we failed to locate intermediate **2**. This was reasonable given the difficulty of the small imidazole ring to stabilize the excess positive charge on the quaternized amine moiety. Instead of the two consecutive proton transfer steps (**1→2→3**) that we initially sought to access **3**, an intriguing double proton transfer (DPT) transition state affording **3** from **1** was identified. **TS 1/3 DPT** yields simultaneous protonation and deprotonation of the amine moiety at 15.7 kcal mol^−1^. Although the adenine ring in **B2** bears an enlarged electronic system compared to **B1**, **2** could not be located for **B2** either. The **B2** ligand system experiences a notably lower barrier at 5.7 kcal mol^−1^ via **TS 1/3 DPT**. Therefore, the existence of **2** in the native environment is presumably highly dependent on the stabilizing factor of the His200 residue via H-bonding. It is advantageous to understand why the barrier decreases in shifting the ligand environment from **B1** to **B2** before going further in our ligand design. Figure 3 shows the key structural parameters for **TS 1/3 DPT** for the **B1** and **B2** ligand systems.

**Figure 4:**
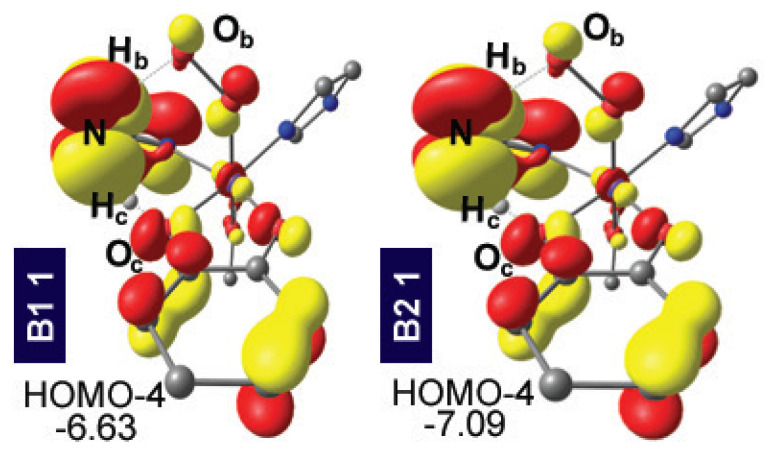
Orbitals hosting the lone pair of nitrogen of the amine moiety of **1** for **B1** and **B2** ligand systems (MO energies are in eV).

The DPT transition state of **B2**, lying 10.0 kcal mol^−1^ lower than the DPT of **B1**, is due to the more weakened O_c_–H_c_ and N–H_b_ bonds. The respective bond lengths at the DPT TS change as 1.184 Å→1.245 Å and 1.101 Å→1.141 Å in switching from **B1** to **B2**. Perhaps more important than these bond lengths, the degree of interaction of the NH_2_ group with the O_b_ and O_c_ centers should be described in relation to the linearity of the O_c_H_c_N and NH_b_O_b_ vectors. Notably, the **B2** framework possesses more linear angles on proton transfer vectors compared to **B1** as a change of 156°→162° for O_c_H_c_N and 161°→171° for NH_b_O_b_ is observed in shifting the ligand framework from **B1** to **B2**. As a result, the **B2** system allows optimal positioning for the amine/ammonium to play its dual basic/acidic role and to interact simultaneously with H_c_ and O_b_. In other words, the nitrogen and hydrogen of the amine moiety in the **B1** system cannot access H_c_ and O_b_ as effectively as those in **B2**. Further evidence of the critical role of the amine moiety is provided by the MOs depicted in Figure 4, where the interaction of the amine lone pair with H_c_ is illustrated.^1^

Inspection of the canonical UDFT frontier MOs reveal that HOMO-4 mainly comprises the nitrogen lone pair of the amine group that is oriented towards H_c_ for both ligand environments. As mentioned above, the O_c_H_c_N vector for DPT TS is more linear for **B2** than **B1**. This should arise from a better electronic interaction of the amine lone pair with H_c_. It is also possible to suggest that the more linear O_c_H_c_N vector yields an enhanced electronic interaction of the amine lone pair with H_c_ and hence the linearization and electronic interaction arise mutually. Even though it is cumbersome, if not impossible, to relate the differences in energy barriers of the transition states of the **B1** and **B2** environments to how the MO that is hosting the amine lone pair is visualized, it is clear that the proper positioning of the lone pair is essential in accessing the DPT TS at low energies.^2^ Thus, it is sensible to assign this orbital interaction pattern the prime importance in obtaining viable barriers. Consequently, we tried to conserve this MO interaction in the later steps of our design where we targeted synthetically more viable systems, as described below.

**Figure 5:**
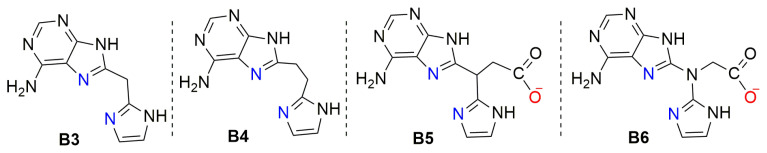
Bidentate and tridentate ligands.

The **B1** and **B2** environments comprised three monodentate ligands at the iron center. Since the realization of such Fe complexes involves experimental complications due to several binding motifs, conformations, and/or the increased possibility of the ligands dissociating, we designed the bidentate (**B3** and **B4**) and tridentate (**B5** and **B6**) environments as outlined in Figure 5.^3^

**Figure 6:**
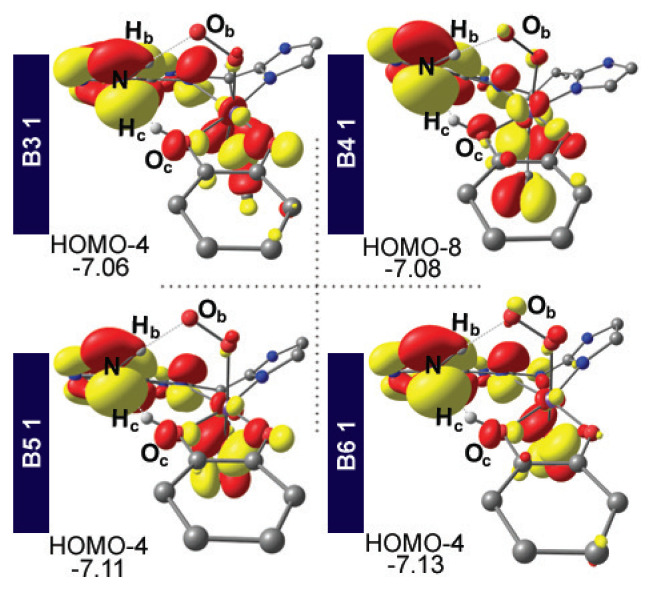
MO interaction leading to proton abstraction from **Cat**. for **1** with ligands **B3**, **B4**, **B5**, and **B6**. Orbital energies are given in eV.

**B4** was designed to investigate the effect of the length and flexibility of the ligand backbone in comparison to **B3**. This is important as the amine group of the adenine moiety is desired to interact with the **Cat**. proton in a fashion similar to that described for **B2**, as described above. In other words, the decreased flexibility of the bidentate ligands compared to the monodentate systems should not obstruct the migration of the **Cat**. proton to the amine moiety. The proton transfer barriers via **TS 1/3 DPT** are essentially identical for **B3** and **B4** at 15.1 and 15.2 kcal mol^−1^, respectively, and both values are higher than that achieved for the **B2** system. The increased barriers are presumably due to the decreased flexibility of the bidentate ligand environments mentioned above (see the Table for selected structural and energetic parameters). Due to the enhanced flexibility of **B4**, the O_c_H_c_N and NH_b_O_b_ vectors for **B4** are more linear compared to **B3**; however, the increase is only marginal. On the other hand, the O_c_–H_c_ bond, whose length correlates with a lower energy barrier, is slightly longer for **B3**. Considering the negligible 0.1 kcal mol^−1^ difference for activation barriers among the bidentate ligand environments, there are no data to suggest that the **B4** system would be superior to **B3**. We surmise that a ligand framework such as **B4** may be useful with a metal having a larger radius than Fe, as will be addressed in future studies. Moreover, the linearization of the aforementioned O_c_H_c_N and NH_b_O_b_ angles and activation barriers are in a slight but significant correlation for **B1**, **B2**, and **B3**; i.e., the more linear the orientations, the lower the 1/3 DPT barrier (Table and Supplementary Figure S10). Consequently, it is not necessary to investigate a longer bridge between the two nitrogen donor rings of the bidentate ligands. Therefore, we continued with the bridge comprising a single carbon atom and targeted an essentially linear arrangement of the O_c_H_c_N and NH_b_O_b_ vectors. Note that important orbital interactions of the bidentate ligands given in Figure 6 show that the targeted MO motif, i.e., the nitrogen lone pair of the amine group orienting towards H_c_, is conserved for **B3** and **B4**.

It is noteworthy that when a functional model from an enzymatic mimic is sought, ease of synthesis is an important issue and utilizing acetate (**B1** through **B4**) with a large degree of lability can be cumbersome. Although glutamate residues in biological systems are used similarly, other means of structural restrictions on the ligands of the first coordination shell are ubiquitous in wild-type enzymes [31]. Consequently, the tridentate ligand frameworks **B5** and **B6** were designed, where acetate is covalently linked to the ligand backbone. Retaining the previously established structural (see the Table) and electronic (Figure 4) features, the proton transfer barriers via **TS 1/3 DPT** were calculated as 5.7 and −0.2 kcal mol^−1^ for **B5** and **B6**, respectively. Note that on the electronic energy surface **TS 1/3 DPT** for **B6** is at 4.4 kcal mol^−1^ and it drops to 0.6 and −0.2 kcal mol^−1^ after the addition of zero-point energies and solvation corrections, respectively. Both corrections suffer from technical drawbacks such as dealing with the imaginary frequency of the transition state structure in quantum chemistry and a proper/realistic definition of the solvent medium in solvation calculations. Further support for the authenticity of **TS 1/3 DPT** for **B6** was obtained by IRC analysis. Nonetheless, the small to absent barrier for **B6** suggests that **3** would be afforded straightforwardly. For the tridentate ligand environments, the **Cat**. protons assume the longest O_c_–H_c_ distances at 1.3 Å in the whole ligand set (Table). Moreover, **B5** and **B6** are the only ligands where the O_c_–H_c_ bond is longer than N–H_c_. Thus, for the tridentate ligands, the **Cat**. proton H_c_, had almost already migrated by the transition state. Inspection of the difference in the bond lengths of O_c_–H_c_ and N–H_c_ show that **B2**, **B5**, and **B6** set possess a **Cat**. proton that migrated to the amine more than the H_c_ of **B1**, **B3**, and **B4** set. Since the former set was obtained with significantly lower DPT barriers than the latter, the extent of the migration of H_c_ correlates nicely with a fairly low DPT barrier (Supplementary Figure S11). The **B2**, **B5**, and **B6** ligand systems thus show a very successful structural design that can reproduce the role of a secondary sphere base in the ring-cleaving dioxygenase enzyme environment. In addition to the extent of migration of H_c_ in the transition state, the linear arrangement of the catechol and amine protons was found to be important at the proton transfer barriers (vide supra). These two critical angles of O_c_H_c_N and NH_b_O_b_ for **B5** and **B6** exceed 160 ° and 170 °, respectively, and are slightly more linear than those for the **B2** and **B3** systems (Table). The orbital interaction pattern that is essential for obtaining viable proton transfer barriers as discussed above for the monodentate and bidentate ligand environments was reproduced for the tridentate ligands, as well. As depicted in Figure 6, the nitrogen lone pair of the amine group was oriented towards H_c_ for **B5** and **B6** and hence the MO pattern we sought was conserved. Thus, the structural, energetic, and electronic features outlined for the tridentate ligand environments **B5** and **B6** suggest that promising realistic candidates for the proton transfer step can be designed judiciously.

At this point, one needs to corroborate the significance of **3** in the reaction mechanism. So far, the ligand systems introduced had shown that the main goal of our current investigation, i.e., proton transfer with a simple ligand design in the initial stages of the HPCD reaction, was in principle possible. On the other hand, at least the synthetically targeted ligand system **B6** should be tested more thoroughly. It is known that the fate of the deoxygenation reaction is bound to existence of **4** as it renders the distal oxygen O_b_, highly reactive [30]. Once the Fe–(O_a_H)–O_b_ moiety is generated, O_b_ attacks the catecholate ring. High reactivity of **4** is very plausible, even without referring to the existing literature, based on simple consideration of the bonding environments of O_a_ and O_b_. In **4**, the proximal oxygen O_a_ poses to have three covalent bonds and hence should immediately lose one of them, namely O_a_–O_b_. Consequently, generation of **4** yields a highly reactive center (O_b_) and the subsequent stages of the reaction are a well-documented Criegee rearrangement [32].

**Figure 7:**
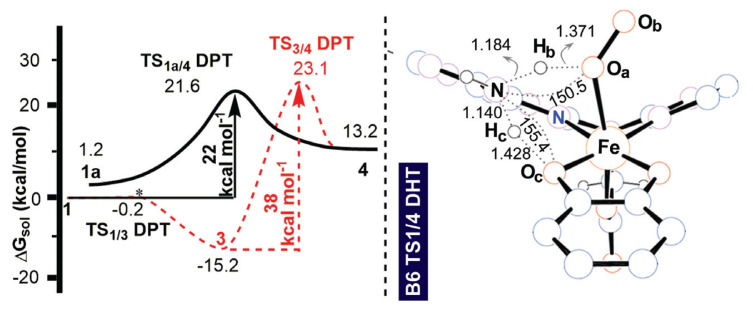
Free energy reaction path and selected structural parameters for **TS 1a/4 DPT** in the **B6** ligand system.

Thus, we investigated the reaction coordinate that yielded **4** with the **B6** ligand environment (Figure 7). The generation of **4** from **3** via a 1,2-proton shift reaction has a large barrier of 38 kcal mol^−1^. Fortunately, however, there is a direct path for generating **4** via a DPT transition state, **TS 1a/4 DPT**, where **1a** is an essentially isoenergetic (at 1.2 kcal mol^−1^) conformer of **1**. **1a** can be accessed from **1** in a barrierless fashion via slight repositioning of the amine and dioxygen fragments (Supplementary Figure S12). **TS 1a/4 DPT** reduces the 38 kcal mol^−1^ barrier to obtain **4** via the 1,2-proton shift to 21 kcal mol^−1^ (see Supplementary Figure S13 for the MO interaction leading to proton abstraction from **Cat**. for **1a**). Since **3** lies in a potential energy well at −15 kcal mol^−1^, once it is accessed it could only return back to the reactant species at room temperature. This result is in excellent agreement with the existing data as previous calculations [13] demonstrated that **3** is a dead end for the reaction. Interestingly, the **B6** system can capture the features that afford the critical intermediate **4** as well as the inhibition of the paths that could originate from **3**. Note that decreasing the 21 kcal mol^−1^ barrier could be possible with modifications to the ligand design. As the current values of the O_c_H_c_N and NH_b_O_b_ angles are 155° and 151°, respectively (Figure 7), more linear proton shuttling vectors should be targeted to further improve the kinetic barrier and this is the goal of a theory-guided synthetic study that is currently underway.^4^ As shown in the recent work of Chatterjee et al. [33], oxidative ring cleavage is possible with a synthetic model of the HPCD core. However, our design herein study is focused on a more general aspect of bioinorganic proton transfer reactions, and in particular how to mimic the secondary sphere effects, which is a prosperous field [34–41].

To conclude, we have presented a theoretical design of the proton transfer steps in the reaction of catecholate monoanion and dioxygen with Fe(II)-centered complexes. The ligand frameworks considered here were built to mimic the early stages of the HPCD mechanism. We have shown that the proton acceptor and donor roles of the secondary sphere histidine in the native HPCD mechanism can principally be achieved by using an amine group as a covalently linked internal base. The role of this internal base was first demonstrated by a simultaneous DPT TS to afford **3** at reasonable activation barriers. It was shown that a linear arrangement of the catechol oxygen, catechol proton, and amine nitrogen is structurally and electronically decisive for the energy barriers and this rationale can be used for designing novel and effective ligand systems. The barrier-lowering interactions of the amine lone pair with the **Cat**. proton can be probed by visual inspection of the relevant Kohn–Sham orbitals. Elongation of the O_c_–H_c_ bond was shown to correlate with a decrease in the proton transfer barrier. A synthetically viable tridentate ligand system **B6** bearing two nitrogen donor sites and a pendant acetate group was suggested for furnishing the most critical species, **4**, which is essential for starting the ring-cleaving events. We believe that [(O_2_)(Cat.-)Fe(II)**B6**] can be furnished as a synthetic model for the HPCD reaction. Synthetic studies, further modifications of the ligand design, and investigations of other transition metals are forthcoming.

## Supporting Information

Figure S1 Structure of the HPCD active site.

Figure S2 Structures studied in the proposed proton transfer path and ligand systems of **B1**–**B6**. **B0** represents the base in the wild-type enzyme.

Figure S3 Perturbations of the reactivity to electronic structures of low (LS)-, intermediate (IS)-, and high (HS)-spin states on complex **1**. The intermediate-spin state was found to be most stable due to enhanced exchange energy. An overall low-spin state restricts the electrons on the Fe center from pairing up, thereby suffering from reduced exchange stabilization, whereas the high-spin state, if maintained throughout the reaction, requires an additional unpairing of lower-lying electrons.

Figure S4 3D representation of structure **1** for **B1**. Side and top views are also shown.

Figure S5 3D representation of structure **1** for **B2**. Side and top views are also shown.

Figure S6 3D representation of structure **1** for **B3**. Side and top views are also shown.

Figure S7 3D representation of structure **1** for **B4**. Side and top views are also shown.

Figure S8 3D representation of structure **1** for **B5**. Side and top views are also shown.

Figure S9 3D representation of structure **1** for **B6**. Side and top views are also shown.

Figure S10 Relation of the double proton transfer barrier to the angle of the two proton transfer vectors for different ligand environments. N-donor ligands separated with a single carbon atom were considered.

Figure S11 Relation of the double proton transfer barrier to extent of migration of the catecholate proton to the amine lone pair.

Figure S12 3D representation of structure **1a** for **B6**.

Figure S13 Relevant MOs of **B6** 1a.

**Table S1:** Energies of the ligand systems studied with the (U)B3LYP/cc-pVTZ//(U)B3LYP/LANL2DZ and (U)M06L-d3/cc-pVTZ//(U)B3LYP/LANL2DZ levels of theory.†

	(U)B3LYP/cc-pVTZ//(U)B3LYP/LANL2DZ					(U)M06L-d3/cc-pVTZ//(U)B3LYP/LANL2DZ				
Str	ΔE small	ΔE large	ΔH	ΔΔG (gas)	ΔΔG (sol)	ΔE small	ΔE large	ΔH	ΔΔG (gas)	ΔΔG (sol)
1	0.0	0.0	0.0	0.0	0.0	0.0	0.0	0.0	0.0	0.0
TS1/3 DHT	11.5	17.5	16.0	18.5	9.8	11.5	23.4	21.9	24.4	15.7
3	−3.8	−0.1	0.0	−0.5	−10.8	−3.8	−4.5	−4.3	−4.8	−15.2
1	0.0	0.0	0.0	0.0	0.0	0.0	0.0	0.0	0.0	0.0
TS1/3	12.2	9.6	7.8	8.4	6.6	12.2	4.4	2.6	3.2	1.3
TS1/3 DHT	2.5	7.5	4.0	4.7	3.6	2.5	9.5	6.1	6.7	5.7
3	−3.2	−7.6	−6.9	−6.9	−7.1	−3.2	−13.7	−13.0	−13.0	−13.2
1	0.0	0.0	0.0	0.0	0.0	0.0	0.0	0.0	0.0	0.0
TS1/3 DHT	7.7	15.1	12.3	14.7	5.4	7.7	24.8	22.0	24.4	15.1
3	2.7	−4.1	−4.0	−4.3	−15.1	2.7	−4.6	−4.5	−4.8	−15.6
1	0.0	0.0	0.0	0.0	0.0	0.0	0.0	0.0	0.0	0.0
TS1/3 DHT	3.9	9.7	6.1	7.7	5.0	3.9	19.9	16.3	17.8	15.2
3	−10.0	−2.6	−2.8	−4.0	−3.4	−10.0	−19.3	−19.5	−20.7	−20.1
1	0.0	0.0	0.0	0.0	0.0	0.0	0.0	0.0	0.0	0.0
TS1/3 DHT	4.2	8.2	4.2	5.3	3.8	4.2	10.1	6.1	7.2	5.7
3	−2.1	−25.8	−25.4	−25.0	−24.9	−2.1	−10.3	−9.9	−9.6	−9.5
1	0.0	0.0	0.0	0.0	0.0	0.0	0.0	0.0	0.0	0.0
1a	1.2	−17.4	−17.3	−17.6	−17.5	1.2	−5.6	−5.4	−5.8	−5.7
TS1a/4 DHT	17.4	17.6	15.3	17.3	13.0	17.4	26.2	23.9	25.9	21.6
TS1/3 DHT	4.2	2.7	−1.1	−0.1	−1.9	4.2	4.4	0.6	1.6	−0.2
3	−0.7	−25.1	−24.8	−24.8	−23.6	−0.7	−16.6	−16.3	−16.3	−15.2
TS3/4	43.4	21.6	18.3	17.5	19.5	43.4	25.1	21.8	21.0	23.1
4	12.0	−7.5	−7.4	−8.1	−8.4	12.0	14.1	14.2	13.5	13.2

† The energies used in constructing the potential energy surfaces were obtained with both B3LYP and M06L (with D3 dispersion) functionals. Both functionals are quite popular and documented to perform well in predicting energies for a variety of chemical reactions. The results of both functionals are in agreement with each other. However, the ΔH calculation for system **B6** with B3LYP yields a negative energy barrier with −1.1 kcal mol^−1^. Although the geometry of the transition state is fully optimized and found to possess one and only one imaginary frequency, the energy calculated with B3LYP is negative. On the other hand, M06L shows this barrier to be about 0.6 kcal mol^−1^. Coupled with the tendency of B3LYP to underestimate the reaction barrier heights in some cases (see [1] Zhao Y, González-García N, Truhlar DG, Journal of Physical Chemistry A, 2005; 109 (9): 2012–2018 and [2] Determan JJ et al., Journal of Chemical Theory and Computation, 2017; 13 (10): 4907–4913) and the general drawback of dealing with imaginary frequencies in TS structures, we decided to continue with the M06L functional instead. We would also like to note that there are no experimental data for the systems studied here to benchmark. As such, the trends in the energies are more informative than the absolute energies. Table S1 shows that the energies of both functionals respond similarly to the geometrical modifications.

**Table S2:** Selected bond lengths and Mulliken spin densities for **B6**.

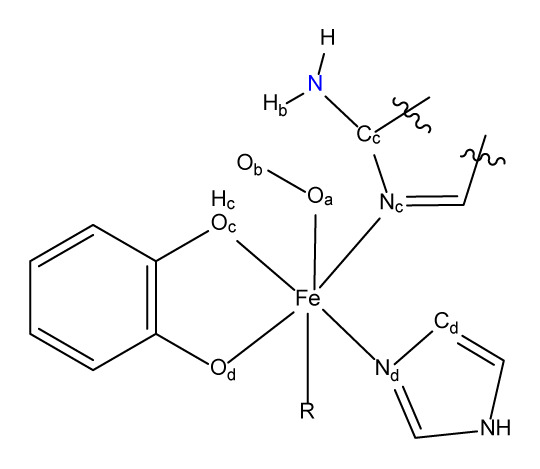						
Bond length/Spin density	1	1/3	3	1a	1a/4	4
Fe–O_a_	2.103	2.232	1.866	2.079	2.284	2.606
Fe–_O_c	2.168	2.114	2.111	2.222	1.931	1.883
Fe–O_d_	1.894	1.939	2.051	1.884	1.836	1.844
O_a_–O_b_	1.363	1.374	1.468	1.370	1.375	1.387
Fe–N_c_	2.269	2.217	2.142	2.235	2.025	2.054
Fe–N_d_	2.121	2.141	2.108	2.114	1.991	1.988
rFe	4.01	3.95	3.08	4.03	2.59	2.63
rO_a_	−0.28	−0.47	−0.18	−0.17	0.38	0.25
rO_b_	−0.52	−0.46	−0.08	−0.62	0.72	0.77

**Table S3:** Selected structural parameters for ligands **B1**–**B6**. Level of theory: (U)B3LYP/LANL2DZ.

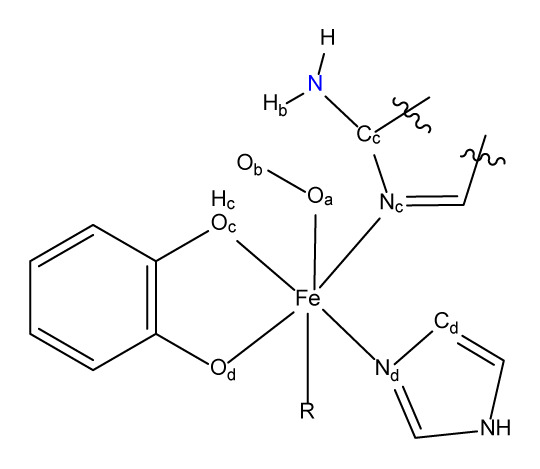																							
	1						1a	TS_1a/4_	TS_1/3_	TS_1/3 DH_						3						TS_3/4_	4
	B1	B2	B3	B4	B5	B6	B6	B6	B2	B1	B2	B3	B4	B5	B6	B1	B2	B3	B4	B5	B6	B6	B6
Fe-O_c_	2.204	2.200	2.171	2.179	2.160	2.168	2.222	1.931	2.225	1.959	2.130	1.949	1.981	2.095	2.114	2.100	2.053	2.032	2.094	2.057	2.111	2.034	1.883
Fe-O_d_	1.914	1.912	1.910	1.857	1.899	1.894	1.884	1.836	1.993	1.851	1.955	1.852	1.837	1.930	1.939	2.080	2.200	2.090	2.134	2.085	2.051	2.102	1.844
Fe-O_a_	2.135	2.101	2.102	2.045	2.108	2.103	2.079	2.284	1.987	2.289	2.218	2.274	2.328	2.254	2.232	1.876	1.850	1.872	1.846	1.859	1.866	1.895	2.606
Fe-N_c_	2.194	2.243	2.249	2.073	2.276	2.269	2.235	2.025	2.236	2.043	2.227	2.043	2.062	2.227	2.217	2.177	2.149	2.179	2.134	2.177	2.142	2.176	2.054
Fe-N_d_	2.118	2.126	2.090	2.245	2.102	2.121	2.114	1.991	2.098	2.022	2.131	1.977	2.097	2.120	2.141	2.085	2.023	2.101	2.110	2.056	2.108	2.030	1.988
O_a_-O_b_	1.369	1.365	1.363	1.362	1.365	1.363	1.370	1.375	1.420	1.375	1.372	1.374	1.376	1.374	1.374	1.463	1.489	1.472	1.484	1.469	1.468	1.629	1.387
O_b_-H_c_	3.110	3.001	3.055	3.019	3.052	3.057	4.087	3.858	1.311	2.913	2.869	2.853	2.852	2.833	2.843	1.005	1.002	0.989	1.003	0.994	0.995	1.309	1.983
O_b_-H_b_	1.696	1.647	1.680	1.649	1.663	1.685	2.947	2.424	1.896	1.514	1.423	1.403	1.322	1.350	1.394	2.763	2.503	3.037	2.856	2.874	3.078	3.922	3.891
N-H_c_	1.618	1.697	1.719	1.819	1.747	1.763	1.952	1.140	3.192	1.313	1.246	1.247	1.262	1.210	1.207	1.962	4.070	4.593	4.291	2.393	2.287	3.156	1.871
O_a_-H_b_	2.470	2.508	2.553	2.534	2.568	2.590	1.822	1.371	2.200	2.273	2.266	2.276	2.172	2.242	2.284	3.178	1.819	2.937	1.848	3.125	2.983	3.123	2.677
O_a_-H_c_	3.010	3.234	3.122	3.304	3.189	3.166	2.827	2.531	2.075	2.934	3.063	3.038	2.986	3.027	3.026	1.936	1.962	1.947	1.956	1.944	1.944	1.101	1.018
O_c_-H_c_-N	152.5	153.0	152.4	142.6	151.3	152.2	145.4	155.4	111.7	155.6	161.9	160.8	162.9	162.1	161.9	67.8	112.2	38.3	46.1	63.1	55.0	53.2	66.8
N-H_b_-O_b_	158.2	174.7	171.9	174.0	173.6	173.3	174.4	170.8	173.9	161.3	171.1	170.5	171.7	172.5	171.8	86.1	133.5	117.1	169.8	101.1	81.8	91.2	79.8
Fe-O_a_-O_b_	131.6	128.4	127.7	124.8	127.0	126.9	115.8	128.2	115.0	131.0	132.2	127.4	130.6	128.4	127.4	119.2	114.6	115.7	116.9	117.2	114.8	113.3	139.9
D(O_c_-Fe-N_c_-C_c_)	29.8	27.1	3.0	15.3	8.4	7.0	22.7	23.3	51.6	27.9	31.3	12.8	21.0	12.6	9.5	39.5	128.8	38.6	31.5	41.9	39.1	29.1	21.5
D(O_d_-Fe-N_d_-C_d_)	58.5	63.9	31.5	20.9	35.5	39.5	35.1	27.0	81.0	52.9	61.5	20.0	22.0	29.2	34.8	90.5	50.6	22.6	1.3	35.5	39.1	42.4	18.4

**Table S4:** Spin densities of selected atoms for ligands **B1**–**B6**. Level of theory: (U)B3LYP/LANL2DZ.

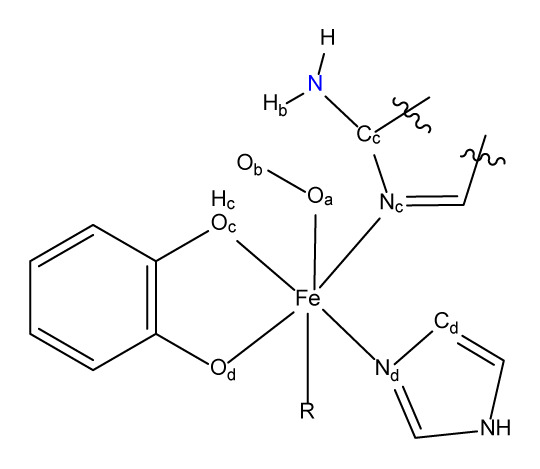																							
	1						1a	TS_1a/4_	TS_1/3_	TS_1/3 DH_						3						TS_3/4_	4
	B1	B2	B3	B4	B5	B6	B6	B6	B2	B1	B2	B3	B4	B5	B6	B1	B2	B3	B4	B5	B6	B6	B6
Fe	4.029	4.033	4.031	2.836	4.020	4.011	4.029	2.592	3.989	2.633	3.980	2.625	2.634	3.970	3.947	3.063	2.828	3.034	2.857	3.017	3.081	3.004	2.632
O_c_	0.066	0.060	0.055	0.041	0.058	0.059	0.047	0.046	−0.027	0.029	0.119	0.032	0.044	0.121	0.125	0.233	0.186	0.237	0.335	0.217	0.248	0.207	0.071
O_d_	0.335	0.327	0.310	0.029	0.328	0.343	0.332	0.096	0.124	0.055	0.370	0.058	0.051	0.373	0.387	0.333	0.388	0.304	0.203	0.358	0.326	0.370	0.092
N_c_	0.060	0.055	0.050	−0.015	0.046	0.046	0.052	−0.010	0.064	−0.012	0.044	−0.013	−0.018	0.042	0.041	0.045	0.057	0.037	0.022	0.047	0.025	0.052	−0.010
N_d_	0.077	0.084	0.083	0.048	0.079	0.073	0.078	−0.009	0.091	−0.004	0.065	−0.014	0.003	0.061	0.054	0.013	0.005	0.035	0.044	0.021	0.043	0.016	−0.011
O_a_	−0.298	−0.293	−0.283	0.424	−0.299	−0.284	−0.172	0.381	−0.036	0.613	−0.449	0.645	0.624	−0.493	−0.472	−0.150	0.025	−0.129	0.014	−0.131	−0.182	−0.057	0.247
O_b_	−0.534	−0.529	−0.521	0.537	−0.519	−0.520	−0.618	0.718	−0.275	0.535	−0.478	0.491	0.463	−0.443	−0.455	−0.078	0.001	−0.057	−0.004	−0.061	−0.076	0.088	0.766
N	0.002	0.001	0.001	0.001	0.001	0.001	0.002	0.003	−0.001	0.001	0.003	0.000	0.001	0.003	0.003	0.001	0.000	0.001	0.000	0.001	0.002	0.001	−0.001
L1	0.059	0.054	0.142	0.035	0.128	0.123	0.133	−0.007	0.065	0.011	0.044	−0.010	0.008	0.112	0.097	0.046	0.070	0.072	0.072	0.075	0.076	0.083	0.004
L2	0.085	0.095							0.103	0.005	0.077					0.024	0.014						
Catecholate Carbons	0.102	0.091	0.087	0.040	0.086	0.100	0.079	0.061	−0.142	0.038	0.216	0.039	0.047	0.202	0.245	0.528	0.515	0.542	0.554	0.520	0.518	0.509	0.065

**Table S5:** Mulliken charges of selected atoms for ligands **B1**–**B6**. Level of theory: (U)B3LYP/LANL2DZ.

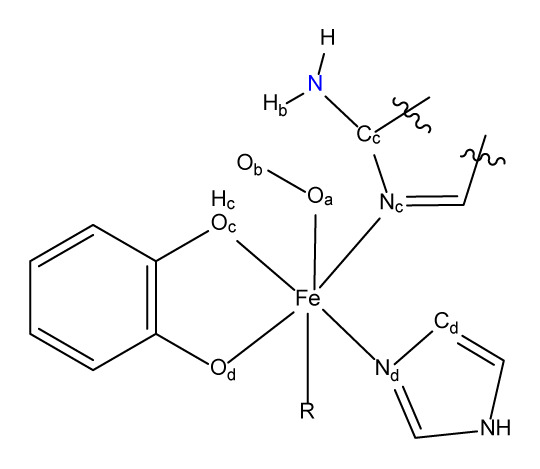																							
	1						1a	TS_1a/4_	TS_1/3_	TS_1/3 DH_						3						TS_3/4_	4
	B1	B2	B3	B4	B5	B6	B6	B6	B2	B1	B2	B3	B4	B5	B6	B1	B2	B3	B4	B5	B6	B6	B6
Fe	0.837	0.822	0.741	0.541	0.804	0.826	0.854	0.687	0.751	0.661	0.770	0.616	0.592	0.765	0.773	0.595	0.517	0.512	0.445	0.546	0.602	0.610	0.718
O_c_	−0.603	−0.615	−0.619	−0.602	−0.628	−0.624	−0.598	−0.610	−0.546	−0.631	−0.624	−0.634	−0.632	−0.633	−0.624	−0.455	−0.457	−0.456	−0.356	−0.470	−0.473	−0.465	−0.582
O_d_	−0.471	−0.477	−0.493	−0.449	−0.490	−0.486	−0.496	−0.439	−0.418	−0.424	−0.431	−0.450	−0.456	−0.450	−0.437	−0.369	−0.330	−0.389	−0.473	−0.353	−0.345	−0.346	−0.454
N_c_	−0.257	−0.237	−0.212	−0.246	−0.176	−0.171	−0.174	−0.215	−0.245	−0.243	−0.241	−0.258	−0.246	−0.194	−0.183	−0.228	−0.250	−0.217	−0.243	−0.213	−0.202	−0.195	−0.229
N_d_	−0.293	−0.292	−0.308	−0.228	−0.283	−0.234	−0.247	−0.214	−0.300	−0.248	−0.288	−0.281	−0.276	−0.271	−0.222	−0.236	−0.262	−0.233	−0.258	−0.263	−0.223	−0.220	−0.222
O_a_	−0.241	−0.249	−0.208	−0.178	−0.214	−0.215	−0.324	−0.347	−0.292	−0.216	−0.237	−0.171	−0.181	−0.191	−0.199	−0.315	−0.329	−0.269	−0.313	−0.271	−0.241	−0.407	−0.303
O_b_	−0.249	−0.229	−0.239	−0.235	−0.243	−0.239	−0.173	−0.177	−0.297	−0.276	−0.253	−0.272	−0.269	−0.264	−0.265	−0.350	−0.360	−0.344	−0.362	−0.338	−0.350	−0.402	−0.114
N	−0.750	−0.728	−0.726	−0.706	−0.719	−0.719	−0.702	−0.749	−0.622	−0.768	−0.759	−0.762	−0.769	−0.763	−0.757	−0.705	−0.631	−0.629	−0.617	−0.667	−0.667	−0.648	−0.714
L1	0.225	0.156	0.410	0.444	0.293	0.209	0.191	0.374	0.107	0.321	0.219	0.546	0.535	0.336	0.264	0.232	0.140	0.361	0.433	0.294	0.165	0.237	0.252
L2	0.219	0.231							0.245	0.256	0.211					0.250	0.274						
Catecholate Carbons	0.417	0.456	0.453	0.484	0.487	0.498	0.483	0.425	0.471	0.442	0.463	0.435	0.487	0.450	0.491	0.517	0.485	0.548	0.545	0.545	0.540	0.559	0.405

## Figures and Tables

**Table:** Activation barriers (ΔG_(sol)_, kcal mol^−1^), critical angles (°), and distances (Å) for TS 1/3 DPT.†

Ligand system	ΔE	Δ O_c_–H_c_–N	Δ N–H_b_–O_b_	R O_c_–H_c_	R N–H_c_	R N–H_b_
**B**1	15.7	155.6	161.3	1.184	1.313	1.101
B2	5.7	161.9	171.1	1.245	1.246	1.141
B3	15.1	160.8	170.5	1.236	1.247	1.157
B4	15.2	162.9	171.7	1.217	1.262	1.199
B5	5.7	162.1	172.5	1.293	1.210	1.191
B6	−0.2‡	161.9	171.8	1.303	1.207	1.166

† Theoretical level: UM06L-D3/cc-pVTZ//UB3LYP/LANL2DZ.

‡ In kcal mol^−1^ ΔE = 4.4; ΔH = 0.6; ΔGgas = 1.6; ΔGsol = −0.2.

## Footnotes

1 Other canonical MOs possessing nitrogen lone pair components also exist at lower energies.

2 We hesitate to interpret the seemingly larger lone pair on **B2** due to problems in comparing MO coefficients in different calculations.

3 Imidazole and acetate are the other two monodentate ligands for the **B1** and **B2** systems in constructing the HPCD active site mimic. The bidentate L–L’ is **B3** or **B4** where acetate is the third ligand. The tridentate Ac–L–L’ is **B5** or **B6** (Figure S2).

4 If the positioning of the amine lone pair is adjusted to achieve a better interaction with the Cat. proton and the O_C_H_C_N vector becomes more linear, a notable enhancement in reactivity might be achieved.
