# Supplementary figures and images for: Homoprotocatechuate dioxygenase active site: Imitating the secondary sphere base via computational design
Source: Turk J Chem. 2023 Sep 30;47(5):1116–24. doi: 10.55730/1300-0527.3598 (PMC10760822; doi:10.55730/1300-0527.3598)

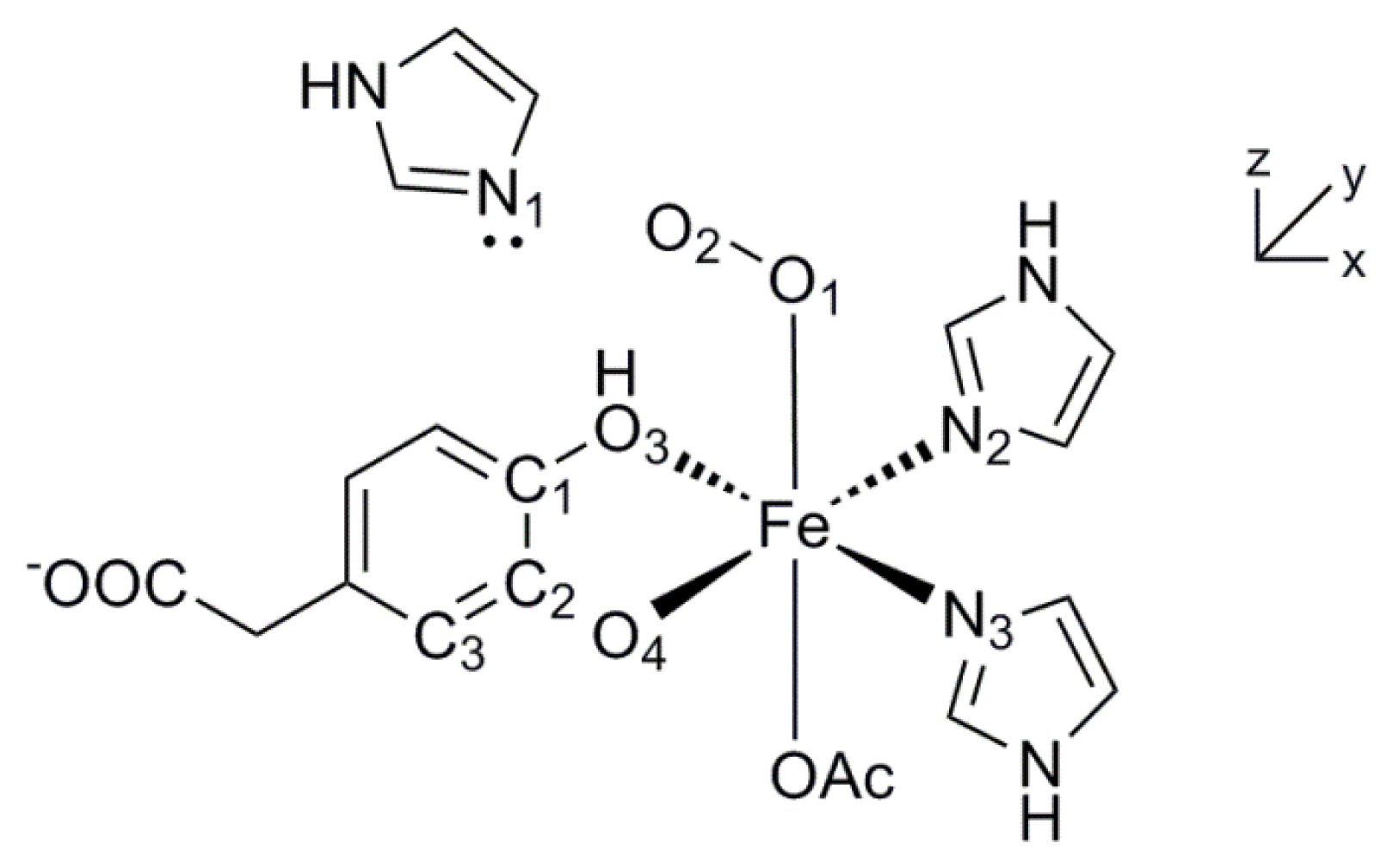

Supplement: Figure S1 — Structure of the HPCD active site. [file turkjchem-47-5-1116s1.tif]

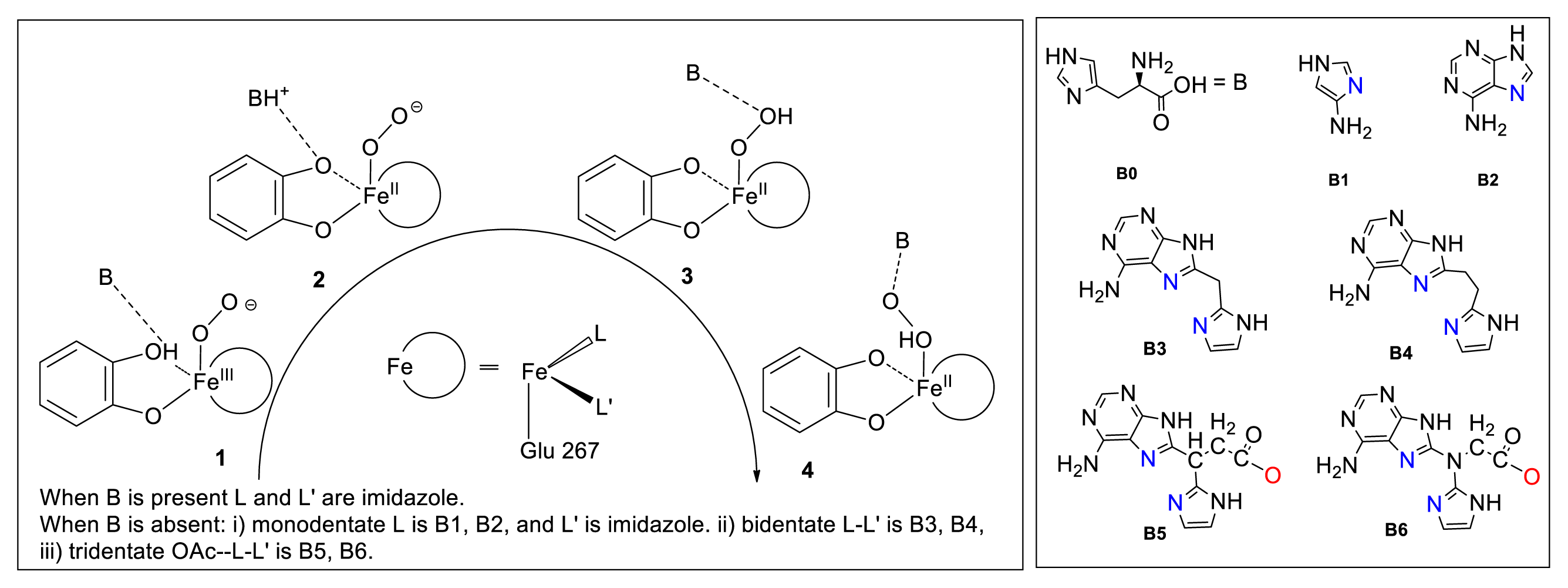

Supplement: Figure S2 — Structures studied in the proposed proton transfer path and ligand systems of B1–B6. B0 represents the base in the wild-type enzyme. [file turkjchem-47-5-1116s2.tif]

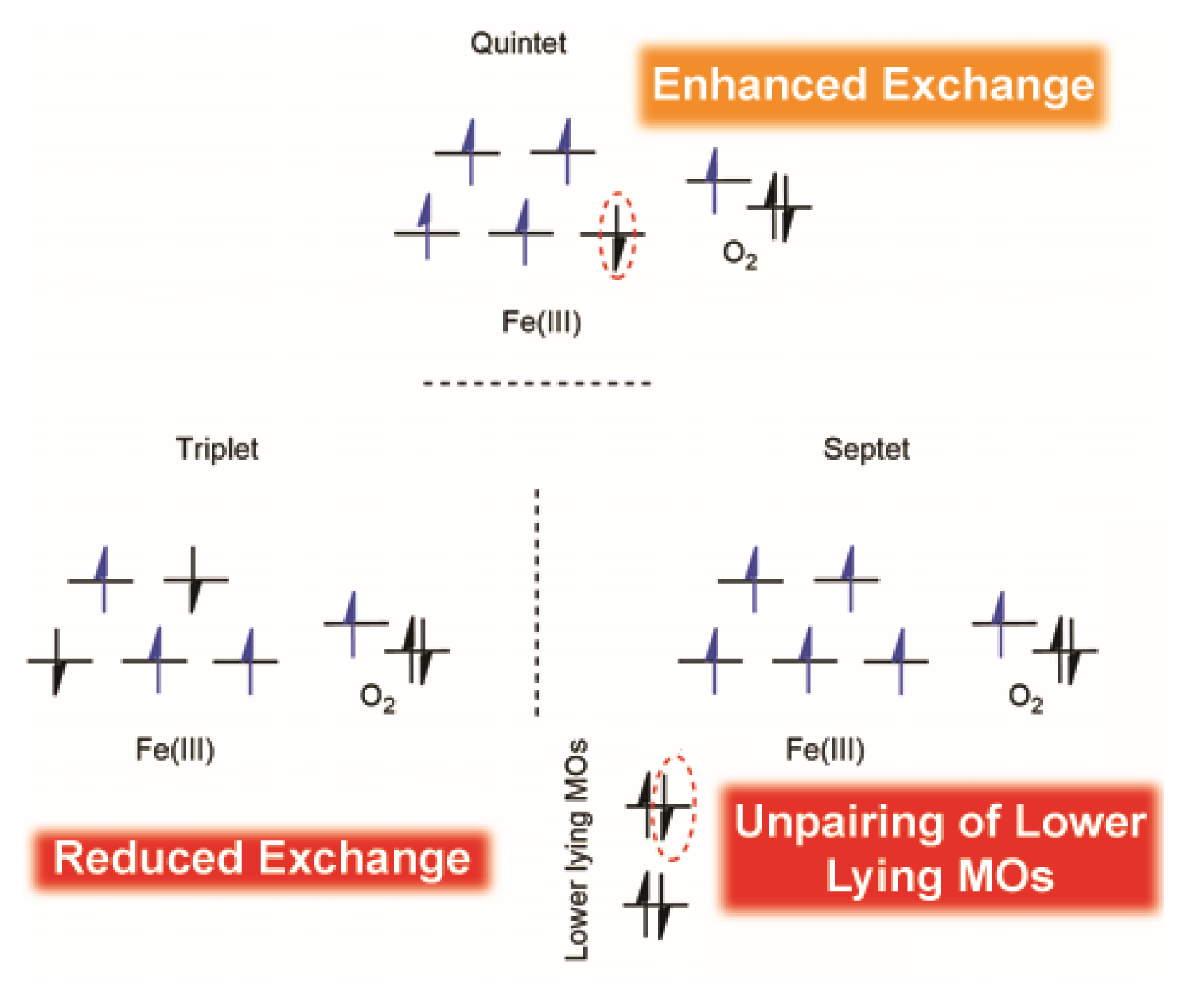

Supplement: Figure S3 — Perturbations of the reactivity to electronic structures of low (LS)-, intermediate (IS)-, and high (HS)-spin states on complex 1. The intermediate-spin state was found to be most stable due to enhanced exchange energy. An overall low-spin state restricts the electrons on the Fe center from pairing up, thereby suffering from reduced exchange stabilization, whereas the high-spin state, if maintained throughout the reaction, requires an additional unpairing of lower-lying electrons. [file turkjchem-47-5-1116s3.tif]

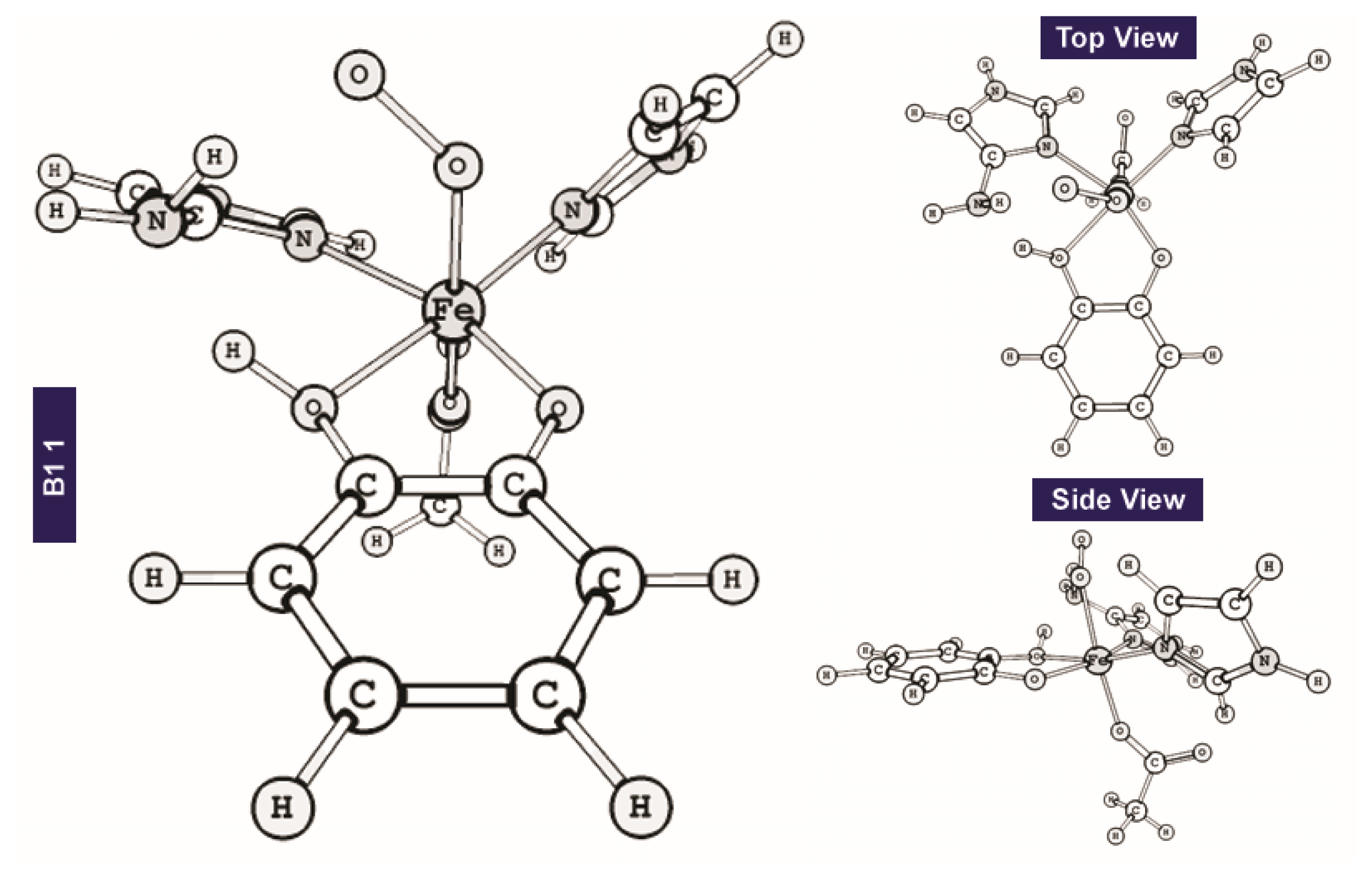

Supplement: Figure S4 — 3D representation of structure 1 for B1. Side and top views are also shown. [file turkjchem-47-5-1116s4.tif]

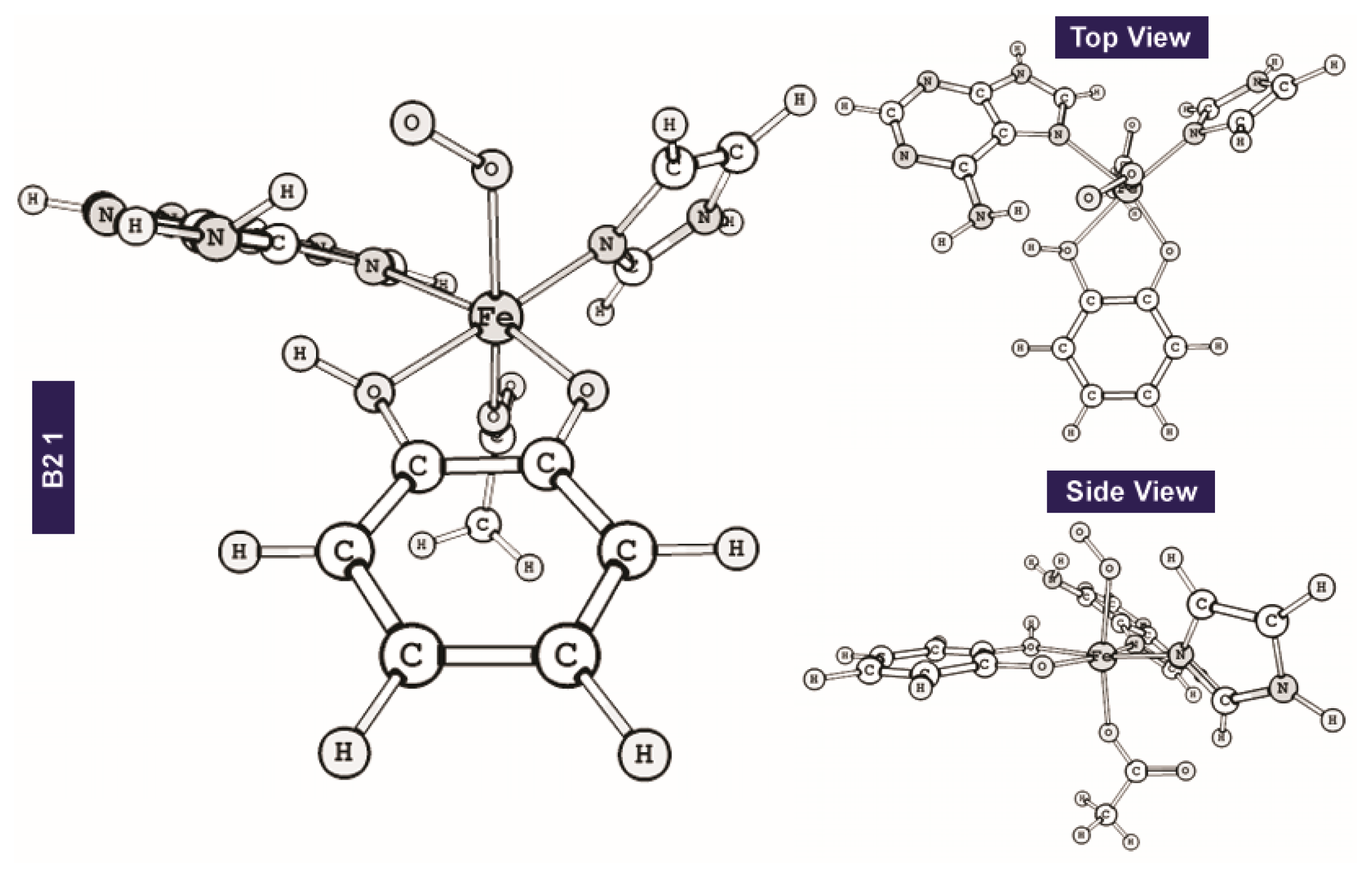

Supplement: Figure S5 — 3D representation of structure 1 for B2. Side and top views are also shown. [file turkjchem-47-5-1116s5.tif]

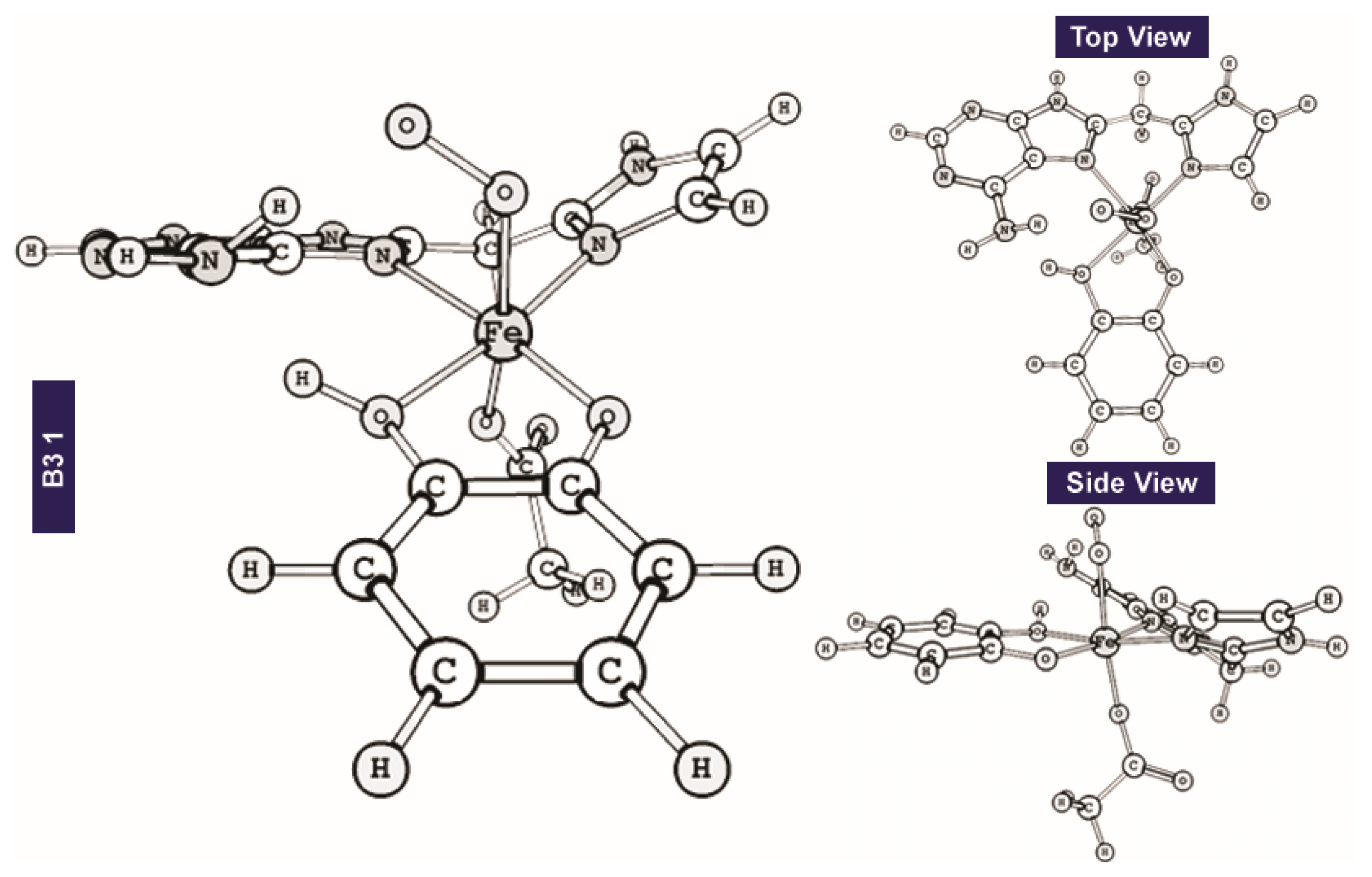

Supplement: Figure S6 — 3D representation of structure 1 for B3. Side and top views are also shown. [file turkjchem-47-5-1116s6.tif]

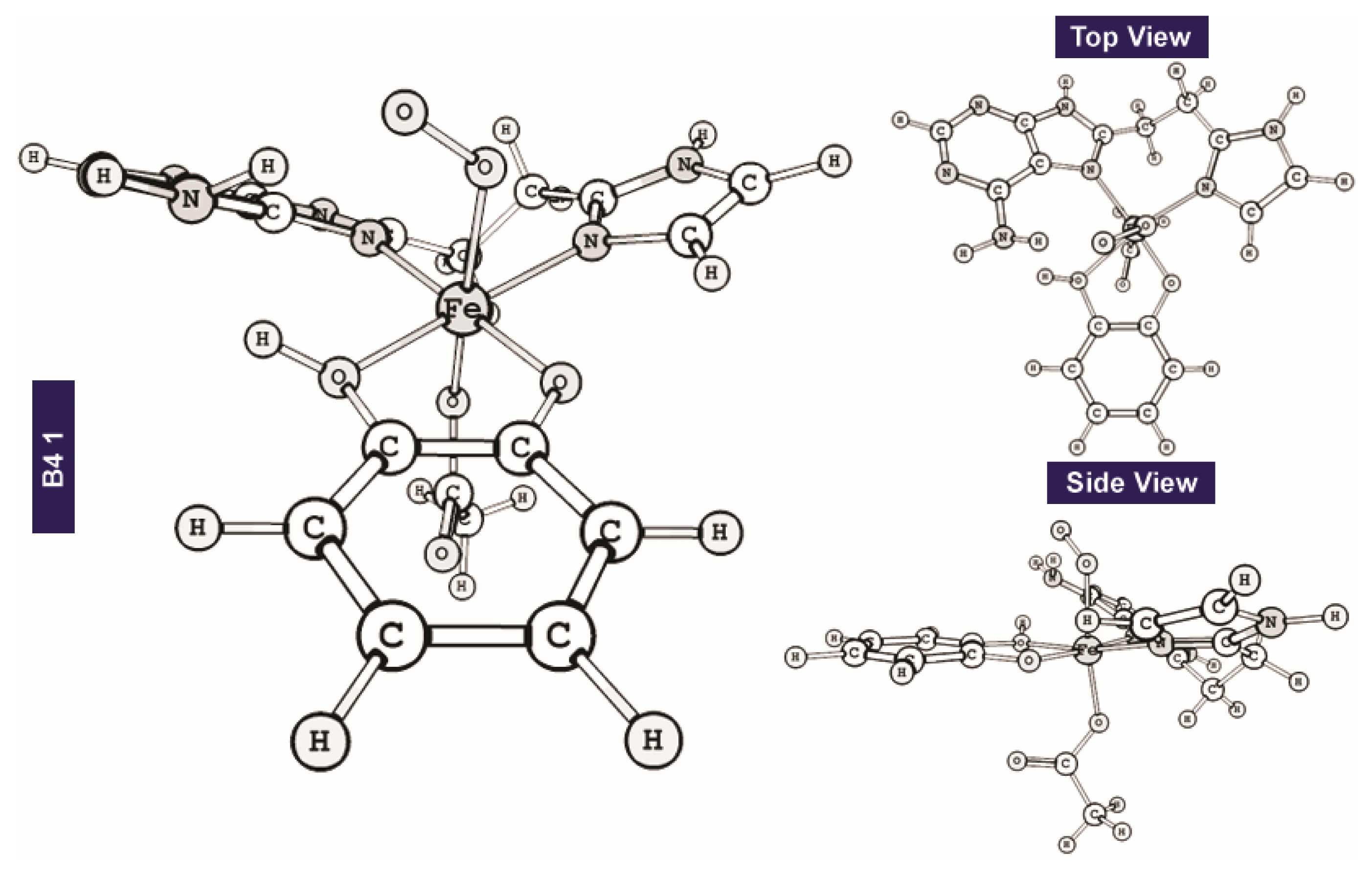

Supplement: Figure S7 — 3D representation of structure 1 for B4. Side and top views are also shown. [file turkjchem-47-5-1116s7.tif]

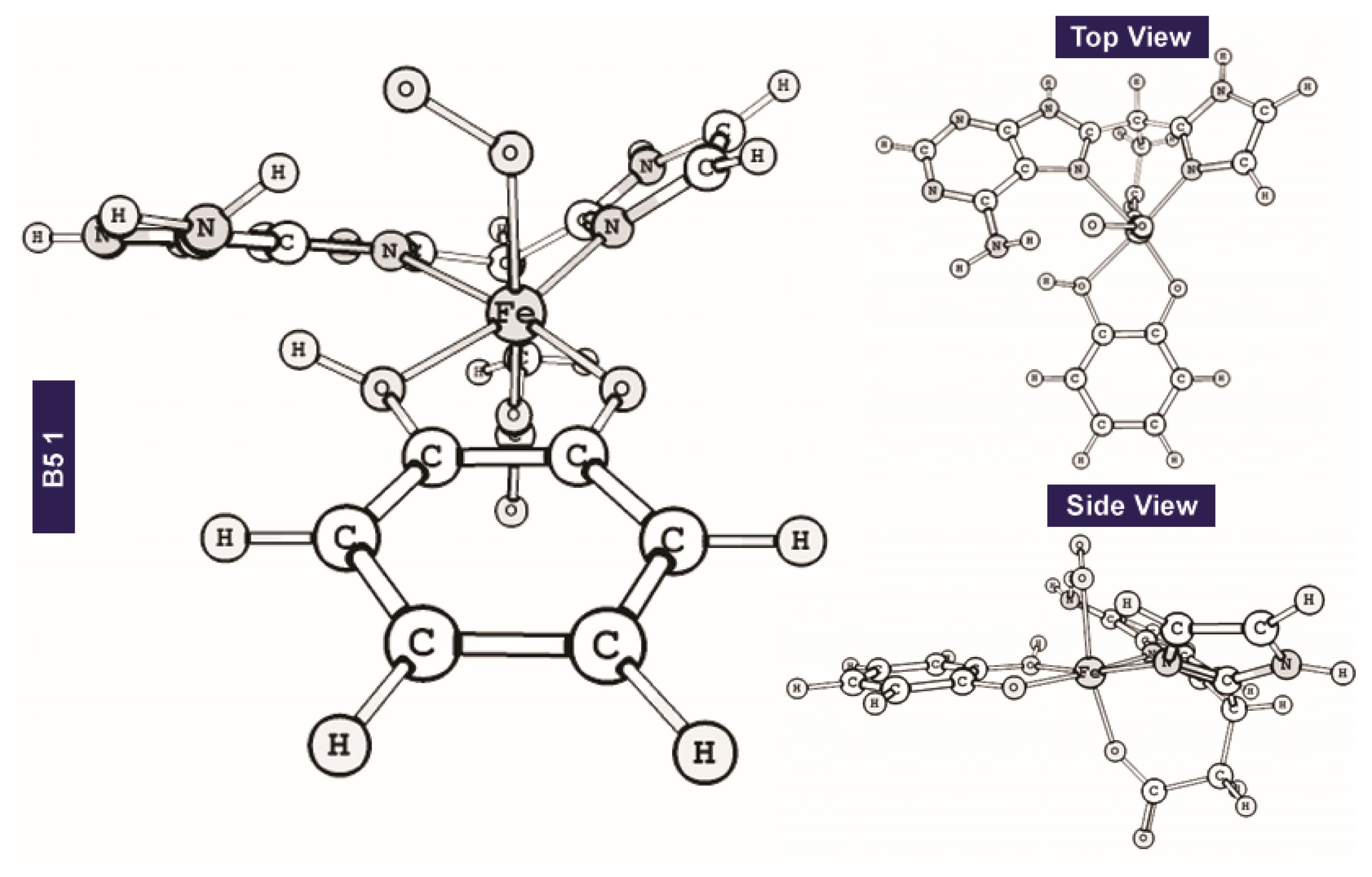

Supplement: Figure S8 — 3D representation of structure 1 for B5. Side and top views are also shown. [file turkjchem-47-5-1116s8.tif]

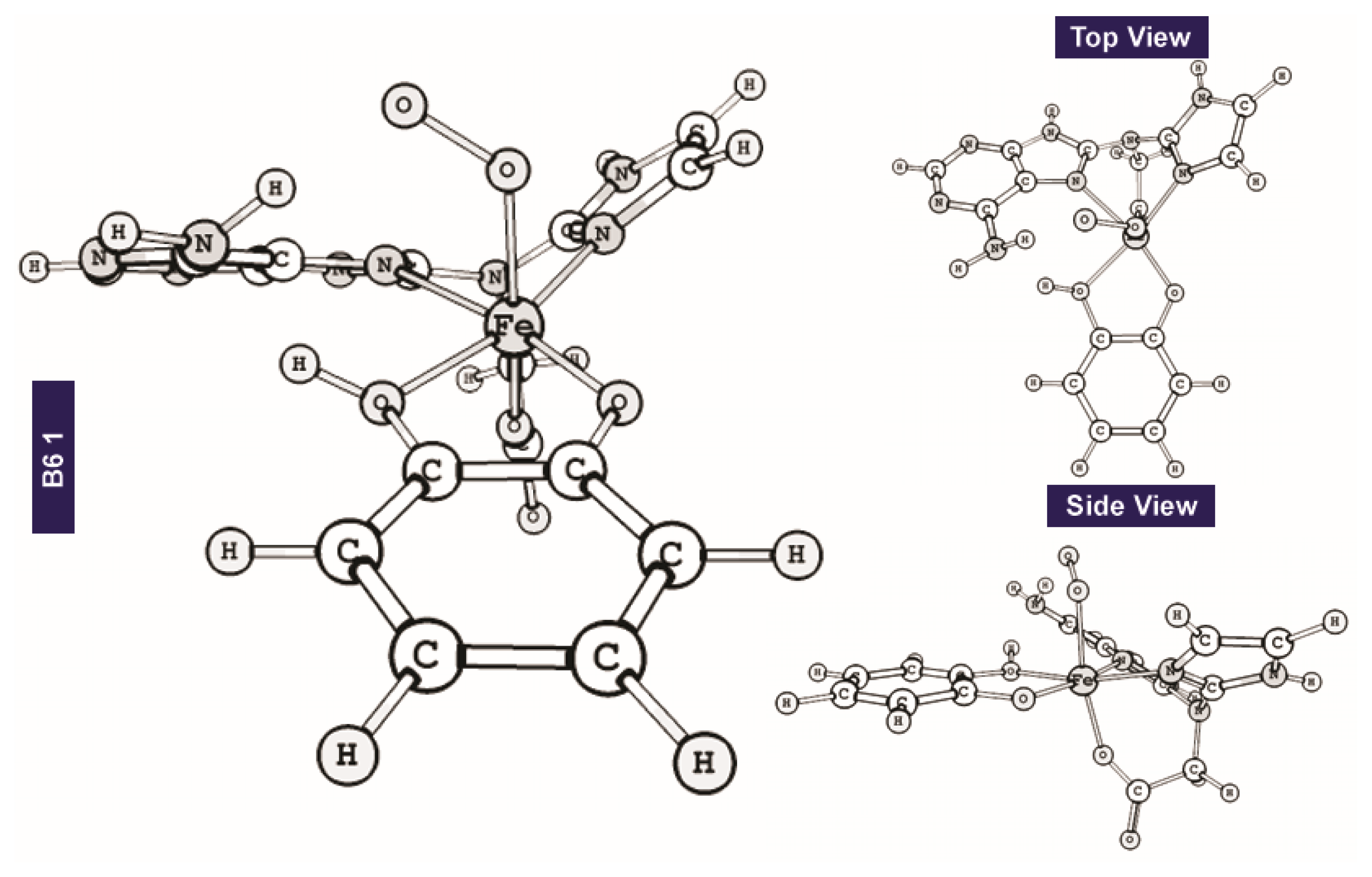

Supplement: Figure S9 — 3D representation of structure 1 for B6. Side and top views are also shown. [file turkjchem-47-5-1116s9.tif]

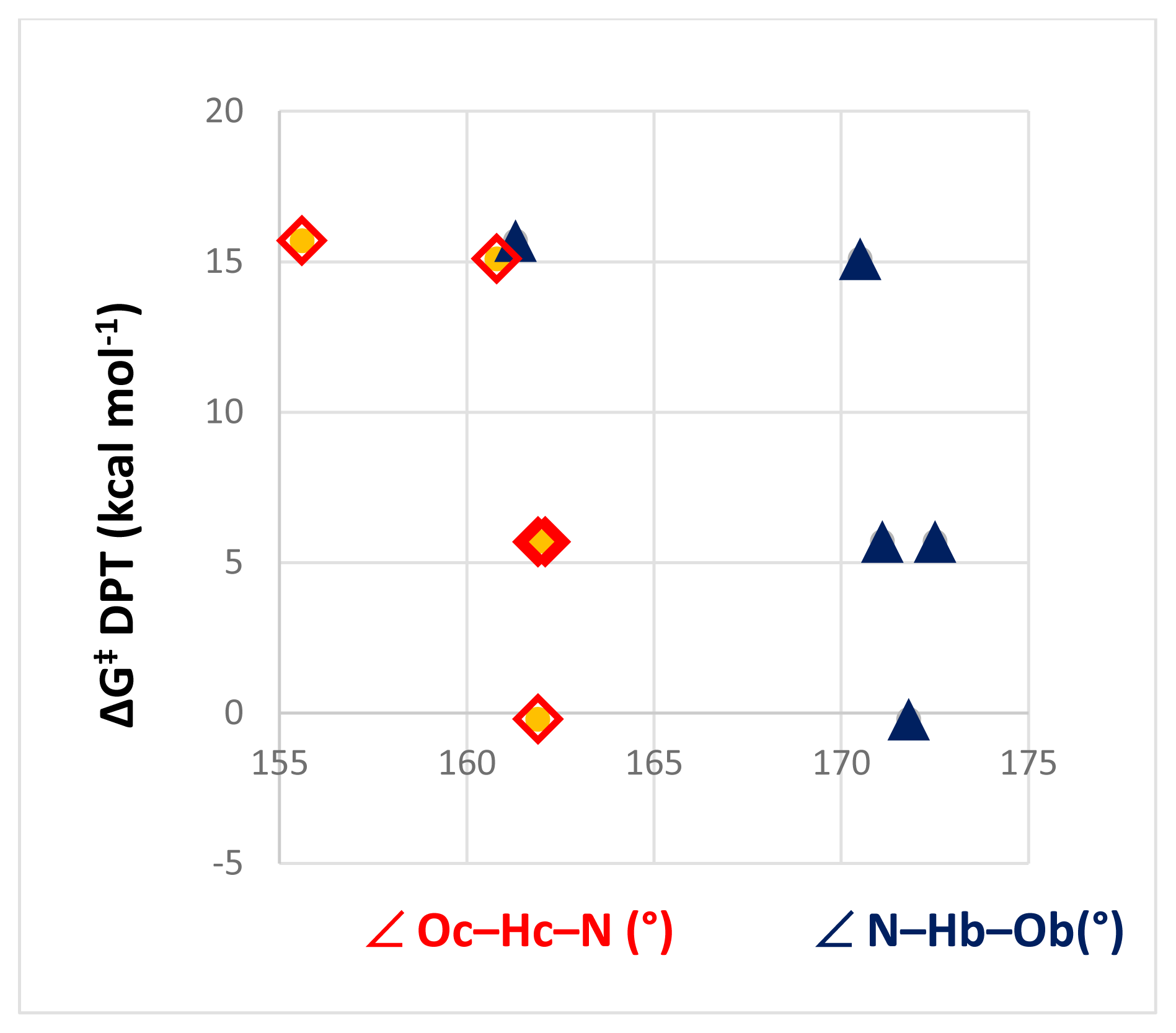

Supplement: Figure S10 — Relation of the double proton transfer barrier to the angle of the two proton transfer vectors for different ligand environments. N-donor ligands separated with a single carbon atom were considered. [file turkjchem-47-5-1116s10.tif]

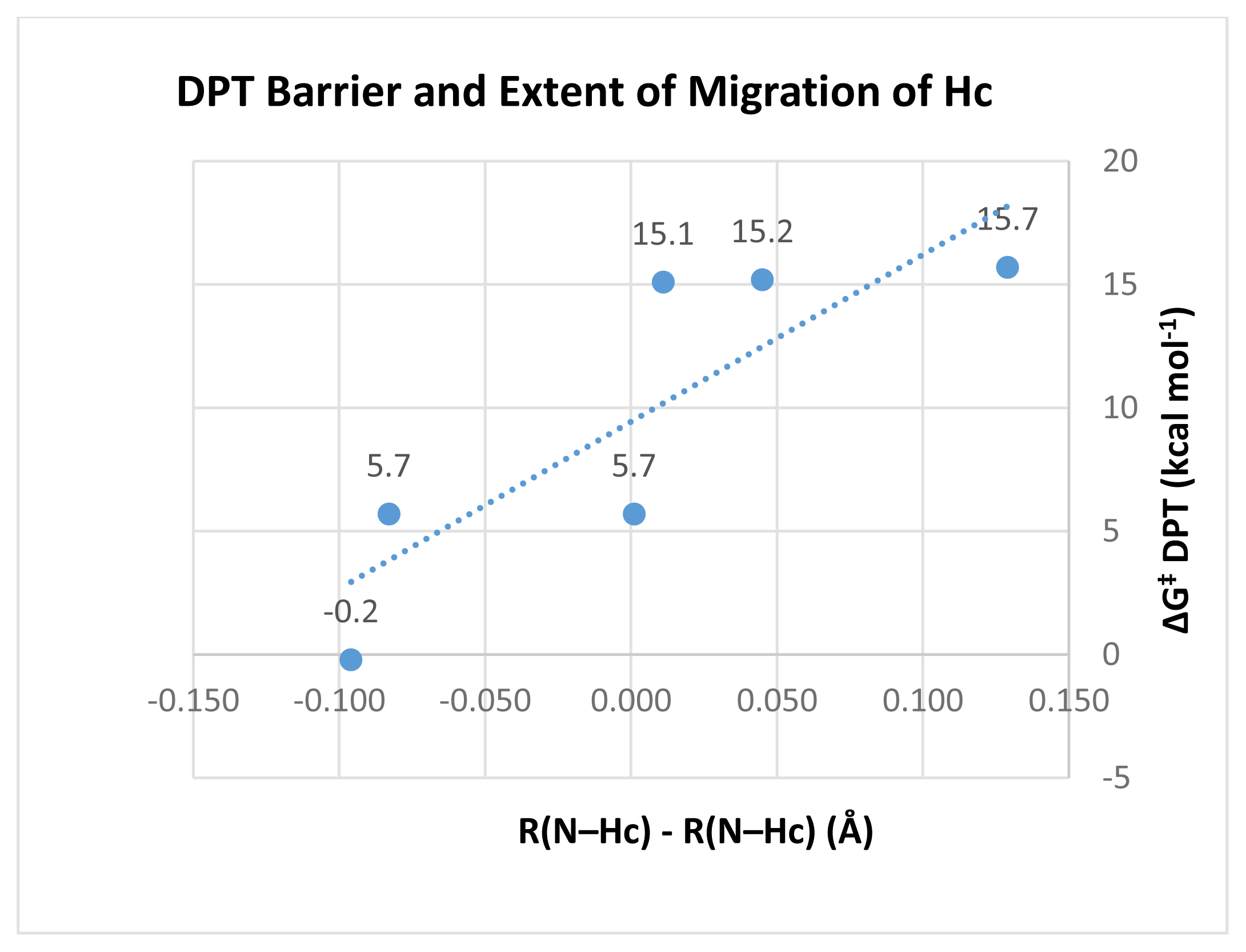

Supplement: Figure S11 — Relation of the double proton transfer barrier to extent of migration of the catecholate proton to the amine lone pair. [file turkjchem-47-5-1116s11.tif]

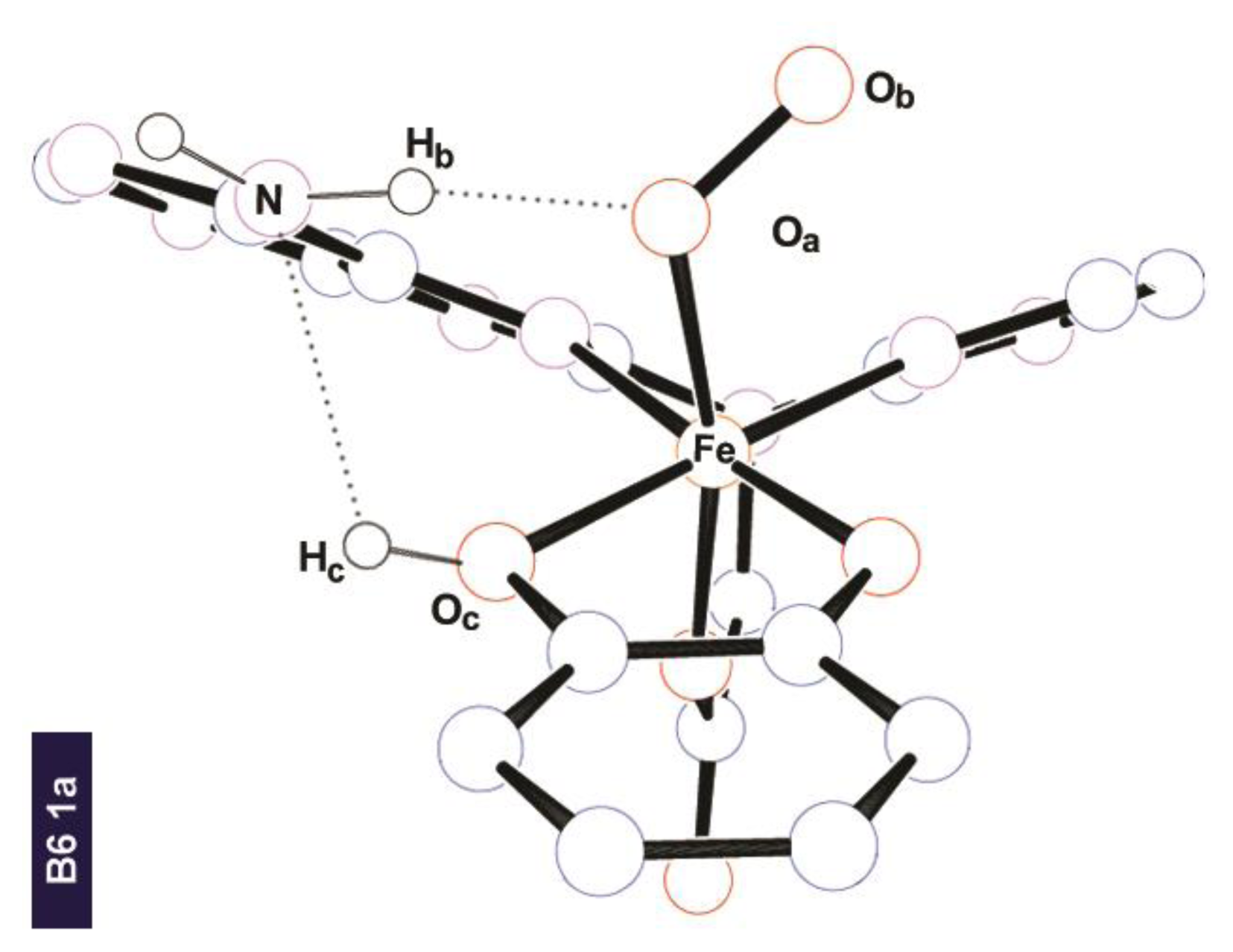

Supplement: Figure S12 — 3D representation of structure 1a for B6. [file turkjchem-47-5-1116s12.tif]

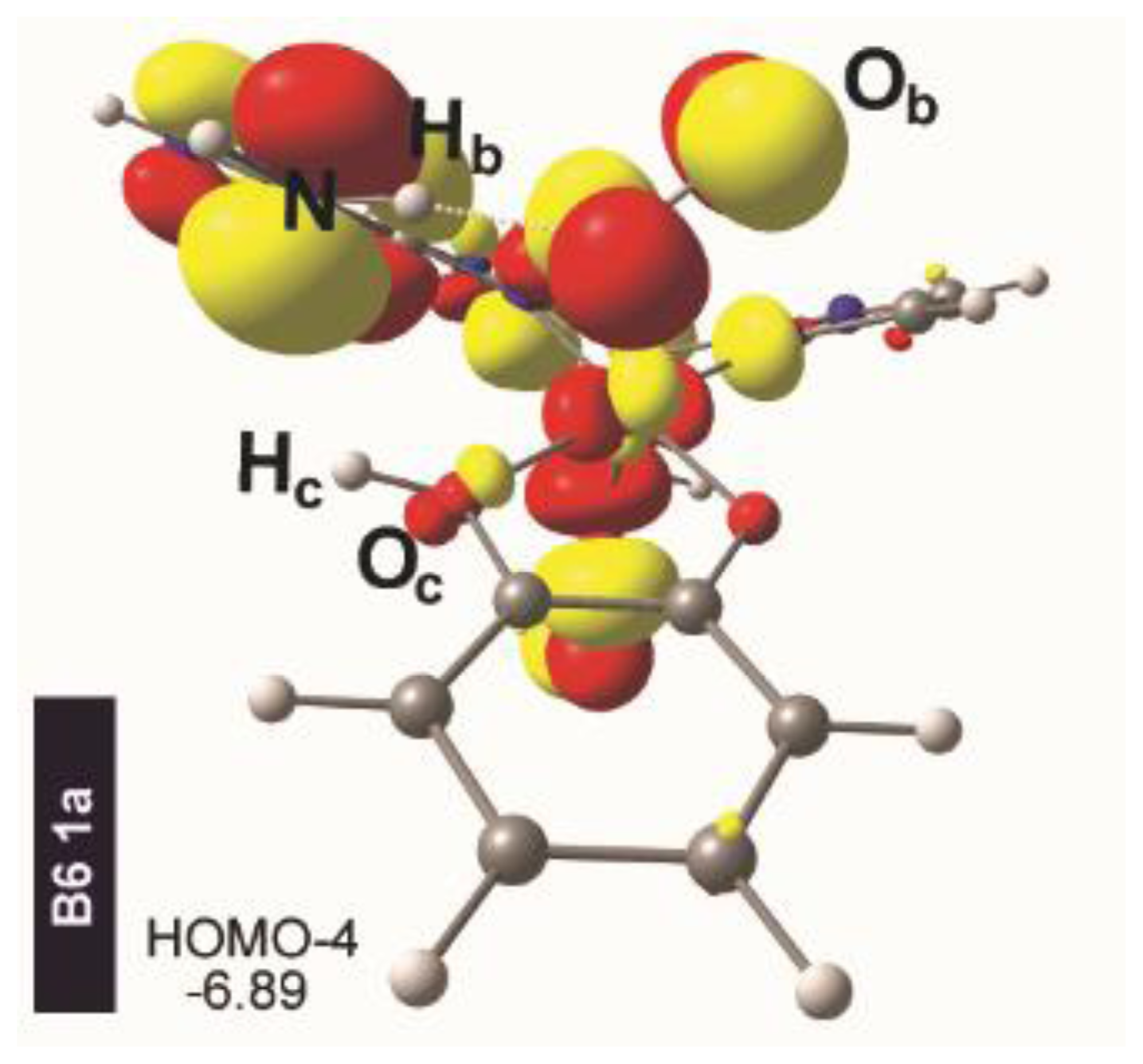

Supplement: Figure S13 — Relevant MOs of B6 1a. [file turkjchem-47-5-1116s13.tif]
